# Malaria Case Fatality Rate among Children under Five in Burkina Faso: An Assessment of the Spatiotemporal Trends Following the Implementation of Control Programs

**DOI:** 10.3390/ijerph17061840

**Published:** 2020-03-12

**Authors:** Mady Ouédraogo, David Tiga Kangoye, Sékou Samadoulougou, Toussaint Rouamba, Philippe Donnen, Fati Kirakoya-Samadoulougou

**Affiliations:** 1 Centre de Recherche en Epidémiologie, Biostatistiques et Recherche Clinique, Ecole de Santé Publique, Université Libre de Bruxelles, 1070 Brussels, Belgium; mady.ouedraogo@ulb.be (M.O.); toussaint.rouamba@ulb.ac.be (T.R.); Philippe.Donnen@ulb.be (P.D.); 2 Institut de Recherche Santé et Sociétés, Faculté de Santé Publique, Université catholique de Louvain, 1200 Brussels, Belgium; 3 Institut National de la Statistique et de la Démographie [INSD], 01 BP 374 Ouagadougou 01, Burkina Faso; 4 Centre National de Recherche et de Formation sur le Paludisme [CNRFP], 01 BP 2208 Ouagadougou 101, Burkina Faso; tigakd@yahoo.fr; 5 Evaluation Platform on Obesity Prevention, Quebec Heart and Lung Institute, Quebec, QC G1V 4G5, Canada; ouindpanga-sekou.samadoulougou.1@ulaval.ca; 6 Centre for Research on Planning and Development (CRAD), Université Laval, Quebec, QC G1V 0A6, Canada; 7 Unité de Recherche Clinique de Nanoro, Institut de Recherche en Sciences de la Santé, Centre National de la Recherche Scientifique et Technologique, 42 Avenue Kumda-Yonre, Ouagadougou, Kadiogo 11 BP 218 Ouagadougou CMS 11, Burkina Faso; 8 Centre de Recherche en Politiques et systèmes de santé-Santé internationale, École de Santé Publique Université Libre de Bruxelles, 1070 Brussels, Belgium

**Keywords:** health programs, malaria, fatality, Bayesian, spatiotemporal

## Abstract

Reducing the 2015 level of malaria mortality by 90% by 2030 is a goal set by the World Health Organization (WHO). In Burkina Faso, several malaria control programs proven to be effective were implemented over the last decade. In parallel, the progressive strengthening of the health surveillance system is generating valuable data, which represents a great opportunity for analyzing the trends in malaria burden and assessing the effect of these control programs. Complementary programs were rolled out at different time points and paces, and the present work aims at investigating both the spatial and temporal pattern of malaria case fatality rate (mCFR) by considering the effect of combining specific and unspecific malaria control programs. To this end, data on severe malaria cases and malaria deaths, aggregated at health district level between January 2013 and December 2018, were extracted from the national health data repository (ENDOS-BF). A Bayesian spatiotemporal zero-inflated Poisson model was fitted to quantify the strength of the association of malaria control programs with monthly mCFR trends at health district level. The model was adjusted for contextual variables. We found that monthly mCFR decreased from 2.0 (95% IC 1.9–2.1%) to 0.9 (95% IC 0.8–1.0%) deaths for 100 severe malaria cases in 2013 and 2018, respectively. Health districts with high mCFR were identified in the northern, northwestern and southwestern parts of the country. The availability of malaria rapid diagnosis tests (IRR: 0.54; CrI: 0.47, 0.62) and treatment (IRR: 0.50; CrI: 0.41, 0.61) were significantly associated with a reduction in the mCFR. The risk of dying from malaria was lower in the period after the free healthcare policy compared with the period before (IRR: 0.47; CrI: 0.38, 0.58). Our findings highlighted locations that are most in need of targeted interventions and the necessity to sustain and strengthen the launched health programs to further reduce the malaria deaths in Burkina Faso.

## 1. Introduction

Malaria remains a major public health issue in sub-Saharan Africa (SSA) and considerably contributes to child morbidity and mortality [1]. In 2018, SSA accounted for 94% of world malaria deaths, of which 67% were children under five [1]. Malaria contributed 5% and 18% of under five deaths worldwide and in SSA, respectively [2]. There is, however, some noteworthy progress that has been made. From 2010 to 2018, the malaria mortality in children under five has approximately halved in the world, and this drop in mortality is attributable to intensified control measures including vector control and drug-based interventions. This drop in mortality was even most marked in SSA [1]. In most SSA countries, this progress is, nonetheless, hampered by the challenge of achieving an early diagnostic and appropriate treatment of malaria cases to prevent the occurrence of complications and death [3].

Burkina Faso is one of the eleven highest-burden countries in the world with a 6% contribution to the 94% malaria deaths recorded in SSA in 2018 [1]. According to the statistics of the Ministry of Health, the same year, malaria was responsible for 53% of the morbidity and 66% of the deaths in children under five seen at health facilities [4]. Like other SSA countries, Burkina Faso implemented several control strategies (preventive and curative) to curb the burden of malaria as the WHO recommendations were being issued. The intermittent preventive treatment in pregnant women (with sulfadoxine–pyrimethamine in replacement of chloroquine) was implemented as of 2005. The same year, the use of artemisinin-based combination therapies (ACTs) for uncomplicated malaria was adopted in lieu of monotherapies. Four years later, the systematic rapid diagnostic testing of suspected malaria cases was implemented to promote a rational prescription of ACT and improve the quality of surveillance data. As of 2010, mass distributions campaigns of free-of-charge long-lasting insecticide-treated bed nets were conducted every three year to achieve universal coverage, the latest having been delivered in 2019. The use of injectable artemisinin derivatives, as alternatives to injectable quinine, for severe malaria treatment was introduced in 2012. The seasonal malaria chemoprophylaxis in children under five was gradually deployed from 2014 to 2019 to achieve full coverage. The indoor residual spraying with long-lasting insecticides, interrupted in 2013, was resumed in 2017 in three health districts out of 70. The IRS was actually being previously conducted in only one health district for three years [5]. Vector control and drug-based preventive measures were provided free of charge contrary to clinical malaria control measures, and this was a potential limitation in the mCFR reduction endeavor. In addition to these measures the activities of community-based health workers were intensified in 2016. The negative effects of the “pay and use health services” policy, in use in sub-Saharan African countries, on populations access to healthcare have been consistently documented [6,7]. As a consequence, the debate on the financing of healthcare has shifted from the “pay and use” to the “free of charge” service approach in favor of children under five and pregnant women who are the most vulnerable populations to malaria [8,9,10,11,12,13,14,15,16]. As of 2016, a free healthcare policy (FHC) for children under five and pregnant women was then launched [17]. Although not specific to malaria, the FHC policy is expected to serve as a catalyst especially for drug-based malaria control interventions by increasing the use of health services. Indeed, quasi-experimental assessments conducted in some sub-Saharan countries found that the free-of-charge healthcare (FHC) policy was effective in increasing access to healthcare especially for the most vulnerable groups. The launch of this policy was the starting point for a free-of-charge care for malaria cases in Burkina Faso.

The analysis of the effect of these overlapping control programs on the trends in malaria indicators is based on routine surveillance data, and for malaria case fatality rate (mCFR), their current exploitation among children under five is limited to the estimation of an unadjusted mCFR [18,19]. The robustness of this kind of analysis is limited since the data used have features that makes classic statistical methods inappropriate. Indeed, classic statistical methods do not allow reliable adjustment for intra-national heterogeneity of malaria case fatality rate. Recently developed and validated hierarchical Bayesian spatiotemporal models were implemented on malaria surveillance data and produced robust estimates [20,21,22,23,24,25,26,27,28]. These models proved useful in addressing complex issues in the data structure.

The present work aims at investigating both the spatial and temporal pattern of malaria case fatality (mCFR) by considering the effect of combining specific and unspecific malaria control strategies.

## 2. Materials and Methods

### 2.1. Study Settings

Burkina Faso is a 272,967 km^2^ landlocked country located in the middle of the West African region and was home to an estimated population of 20 870 060 inhabitants in 2019 [29]. The health system has three levels [4]. On the administrative viewpoint, from top to bottom, we have the central level (global data processing, decision-making), the intermediate level (regional coordination and supervision) with 13 regional health directorates and the district level (field operations) with 70 health districts [4]. From the technical viewpoint, the bottom has two layers consisting of 1896 public primary health facilities that refer to 45 district hospitals with surgical capacity. Those district hospitals, in turn, refer to the eight regional hospitals, which themselves, refer to the six teaching hospitals, which are the third level [4].

The climate is tropical with a single four month-long rainy season from June to September. The health districts located in the southwestern part of the country have a higher annual rainfall (above 950mm) compared with those located in the northern part (599–715 mm) [30]. The transmission of malaria is generally stable and occurs throughout the year with a peak in the rains. *Plasmodium falciparum* is responsible of more than 90% of the infections [31]. The percentage in 2014 of the population living under the poverty line was estimated at 40.1%, with regional variations from 9.6% in the Centre Region to 70.4% in the North Region [32]. This rampant poverty exists alongside a low-performance health system with high mortality rates (129 for 1000 children in 2010) [33]. In addition, the proportion of caregivers promptly seeking (within 24 h of onset) healthcare for feverish children is still low in the Sahel (28%), Boucle du Mouhoun (31%) and Southwest regions (42.8%) [34]. The performance rate of the malaria rapid diagnostic test (mRDT) is also low in the Boucle du Mouhoun (30.3%), Sahel (40.1%) and Hauts Bassins (37.9%) regions [34]. In 2017, the percentage of uncomplicated malaria cases treated with ACT among feverish children for whom care was sought at public primary health facilities was 79% overall [34].

### 2.2. Data Sources

#### 2.2.1. Malaria Case Fatality Data

The monthly count data on severe malaria cases (confirmed and presumed cases) and malaria deaths in children under five from January 2013 to December 2018, aggregated at district level, were extracted from the national health statistics repository (ENDOS-BF), a DHIS2 (developed by Health Information Systems Programme, University of Oslo, Norvège) web-based platform operational as of 2013 [35]. This data repository has substantially improved the quality of the data by improving its timeliness and completeness. The data discrepancy index between the source and the reported data improved from 61% in 2013 to 80% in 2018 [36].

#### 2.2.2. Malaria Control Programs Data

To assess the impact of the FHC policy [17] on mCFR in children under five, the period from 2013 to 2018 was considered and split into two time intervals: before FHC policy (from 2013 to 2015) and after FHC policy (from 2016 to 2018). This exposure variable was defined as a binary one: 0 for “before FHP” and 1 for “after FHP”.

The data on the availability of mRDT and ACT at health facilities were extracted from the results of the Service Availability and Readiness Assessment (SARA) surveys conducted in 2012, 2014 and 2016 [37]. These SARA surveys, sponsored by the Ministry of Health, are cross-sectional surveys that collected data on a random sample of at least 687 health facilities each year [38]. The health facilities were selected using a stratified two-stage cluster sampling method based on the administrative division of the country in regions.

The data on malaria case management with ACT were extracted from the results of the Malaria Indicators Surveys (MIS) conducted in 2014 and 2017. The MIS, sponsored by the National Statistics and Demography Institute (INSD) in collaboration with a global consulting company (ICF International), are cross-sectional surveys that collected data on a random sample of 6552 and 6332 households in 2014 and 2017, respectively [34,37]. The households were selected using a stratified two-stage cluster sampling method based on the admirative division of the country in regions.

#### 2.2.3. Control Variables

Acute malnutrition is a debilitating condition that impairs the immune response to pathogens [39], including malaria parasites and, even more, the response to some antimalarial drugs [40]. It has been linked to an increased risk of childhood death [41]. At population level, the burden of acute undernutrition in children under five is best measured by the prevalence of global acute malnutrition (GAM), which informs on their short-term nutritional history as well the general population nutritional status. The GAM index is classified into five categories from very low to very high, and an emergency threshold was set at 10% to indicate public health immediate actions exigency [42]. We have then adjusted our model with a dichotomous variable for GAM, based on the 10% UNHCR standard threshold (0 if GAM < 10%; 1 if GAM ≥ 10%).

Two additional variables, the distance to the nearest health facility (binary: 0 for “< 5 km”, 1 for “≥5 km”) and the number of nurses for 5000 inhabitants (binary: 0 for “< 5000”; 1 for “≥5000”), were derived from health indicators (mean theoretical health facility operational range and number of inhabitants for 1 nurse, respectively) in the health statistics annual yearbooks of 2013 to 2018 [4,18,19,43,44,45]. These two variables were recoded according to the WHO standards [46].

The geographic location of the health districts and the year were used to merge the different datasets.

### 2.3. Statistical Analysis of Malaria CFR

#### 2.3.1. Estimating District-Level Interventions Coverage

The data on the confounders of interest (mRDT and ACT availability, ACT use), obtained from the MIS and SARA surveys, were collected from a random sample of health districts to derive estimates at regional and national levels. These data were, therefore, not appropriate to compute reliable estimates for each of the 70 health districts. To address this issue, we fitted a Bayesian binomial model to the aggregated data on these confounders to estimate them at the district level. The details on the modelling of the estimates at the district level are provided in the Appendix A1.

#### 2.3.2. Descriptive Analysis of Temporal Trends

The malaria case (severe) fatality rate was estimated by dividing the monthly total number of malaria deaths in children under five by the monthly total number of severe malaria cases in children under five. This estimation was done for each health district [47]. A time plot was drawn to describe the temporal evolution of the crude mCFR in Burkina Faso on a monthly basis between January 2013 and December 2018.

#### 2.3.3. Spatiotemporal Modelling

The occurrence of malaria death in children under five on a monthly basis at the health district level was a rare event. Moreover, we fit spatio-temporal modeling with zero-inflated Poisson (ZIP) and negative binomial (ZNIB) distribution and used the deviance criterion (DIC) to determine the best spatio-temporal model. Results (Appendix A2) indicate a Bayesian ZIP regression model fit the data to describe the spatiotemporal evolution of mCFR in children under five living in Burkina Faso [48]. If $y_{\mathrm{it}}$ and $n_{\mathrm{it}}$ represent the number of malaria deaths and the number of severe malaria cases in children under five, respectively, in the health district *i* = (1, 2, …, 70) at time t = (1, 2, …, 12), the malaria deaths can be modelled as follows:

(1) $$y_{it}∼ZIP(n_{it}{µ}_{it})$$

The risk of death ${µ}_{it}$ can be modelled as follows:

(2) $$\log\left(\mu_{\mathrm{it}}\right)=\propto +s_{i}+v_{t}+X^{T}\beta +\delta_{\mathrm{it}}+\varepsilon_{\mathrm{it}}$$

where:

β is the regression coefficient,

$s_{i}$ is the spatial variance,

$v_{t}$ is the temporal variance,

$\delta_{it}$ space-time interaction.

A non-informative Gaussian prior distribution is attributed to the vector of the regression coefficient β (β*∼* N(0, 1000)) [48].

The term $\delta_{it}$ allows for each health district to have its own trend. This last term indicates the additional variability in the data, not explained by the other components of the model. For the spatial random effect term $s_{i}$, we assign the Besag–York–Mollié (BYM) model [49]. This spatial variance was decomposed into the sum of a structured spatial random effect ($u_{i}$) and an unstructured spatial random effect $\left(\zeta_{i}\right)$. The combination $u_{i}+\zeta_{i}$ accounts for the spatial dependency in the modelling process. $\zeta_{i}$ follows a Gaussian distribution, and $u_{i}$ is modelled via a conditional autoregressive regression model (CAR) with a neighbor matrix W of size N × N, where the diagonal component $w_{ii}=0$ and the non-diagonal component $w_{ij}=1$ if districts i and j share a common border, and $w_{ij}=0$ otherwise. The prior posterior CAR distribution, defined for the spatial random effect, assumes that adjacent districts tend to have similar risks of mCFR in children under five. The temporal variance $v_{t}$ was decomposed into a sum of a structured temporal random effect $(\gamma_{t}$) modelled by a AR (1), that is $\gamma_{1}\sim N\left(0,\frac{\sigma^{2}}{1-\rho^{2}}\;\right)\;and\;\gamma_{l}\sim N\left(\rho \gamma_{l-1},\sigma^{2}\;\right),\;l=2,\ldots ,67$, with the autocorrelation parameter ρ quantifying the degree of dependency between three consecutive months, and an unstructured temporal random effect $\left(\phi_{t}\right)$ modelled by a Gaussian $\phi_{t}∼N\left(0,\frac{1}{\tau_{\phi }}\right)$ with an overdispersion parameter $\epsilon_{it}∼N\left(\;\sigma_{\epsilon }^{2}\;\right)$. As suggested by Gelman et al., we assume that all the standard deviations of random effects $\frac{1}{\tau_{\phi }}\;and\;\sigma_{\varepsilon }$ follow a strictly positive prior semi-Gaussian distribution $N_{+\infty }\left(0,10\right)$ [50].

The Integrated Nested Laplace Approximation (INLA) package in R was used to implement the models, which yielded an a posteriori distribution of the marginal effects of the model parameters and the interventions effects. These parameters were summarized using the case fatality rate risk (CFR) and the 95% credible interval. The Richardson classification [51] was used to estimate the excess risk and classify the health districts into three categories according to the national malaria control threshold in year 2018 (1.01%) as follows: (a) high risk of mCFR if the posterior probability distribution for a child to die from severe malaria is above 0.8, (b) low risk if the probability is below 0.2 and (c) moderate risk if the probability is between 0.2 and 0.8. The results (risk of mCFR in children under five) were exported to and mapped with QGIS version 2.18.14 (developed by Open Source Geospatial Foundation, Chicago, IL, USA) [52]. The deviance information criterion (DIC) was used to compare the null model with the full model, and the model with the smaller DIC (best fitting model) was kept.

#### 2.3.4. Ethical Considerations

The present study used data collected from public health facilities and recorded in the national health data repository (ENDOS-BF), which was accessed through the directorate in charge of the national health statistics management (DGESS) with the authorisation of the Ministry of Health. An ethical approval was not requested because the data used were aggregated at district level with no personal information accessible.

## 3. Results

### 3.1. Summary of Crude Estimates of Severe Malaria Case Fatality Rate in Children under Five

**Figure 1:**
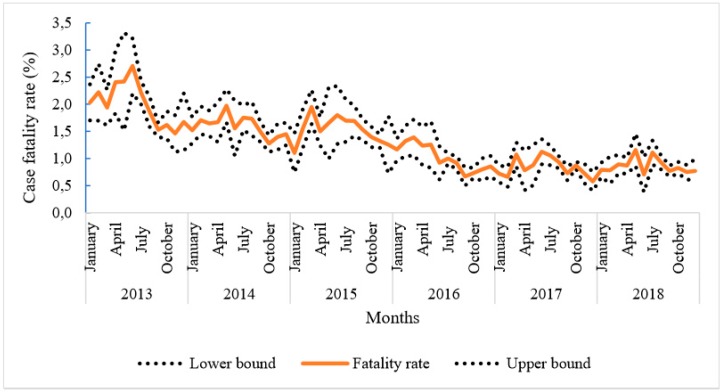
Evolution of malaria case fatality rate in children under five from 2013 to 2018.

There was a decreasing trend in mCFR between 2013 and 2018. Indeed, the mCFR went from 2.0 (95% IC 1.9–2.1%) to 0.9 (95% IC 0.8–1.0%) deaths for 100 severe malaria cases in 2013 and 2018, respectively, that being a more than 55% drop (Figure 1). However, it was noticeable that the decrease appeared to stall from September 2016 onwards.

### 3.2. Spatio-Temporal Dynamics of Crude and Predicted Rate of Malaria Case Fatality Rate by Quarter and by District

**Figure 2:**
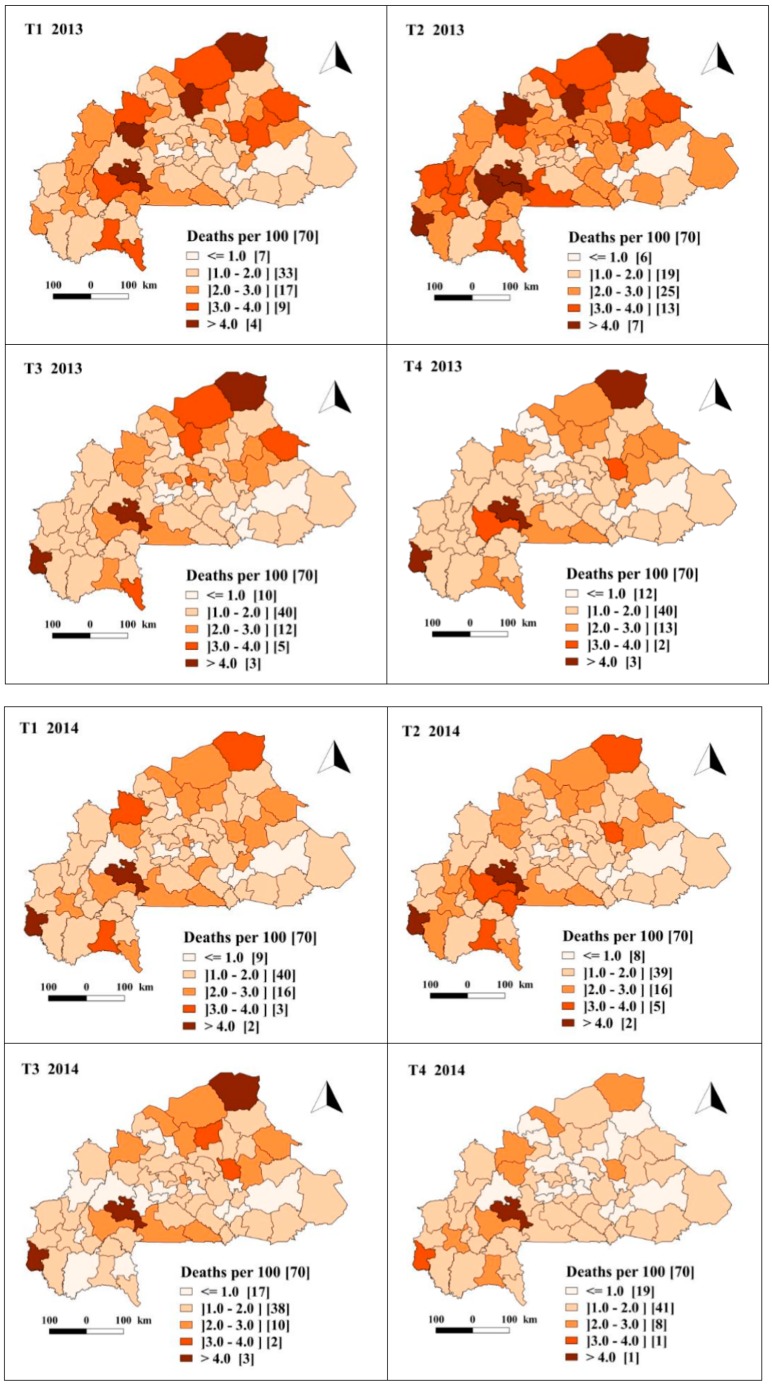

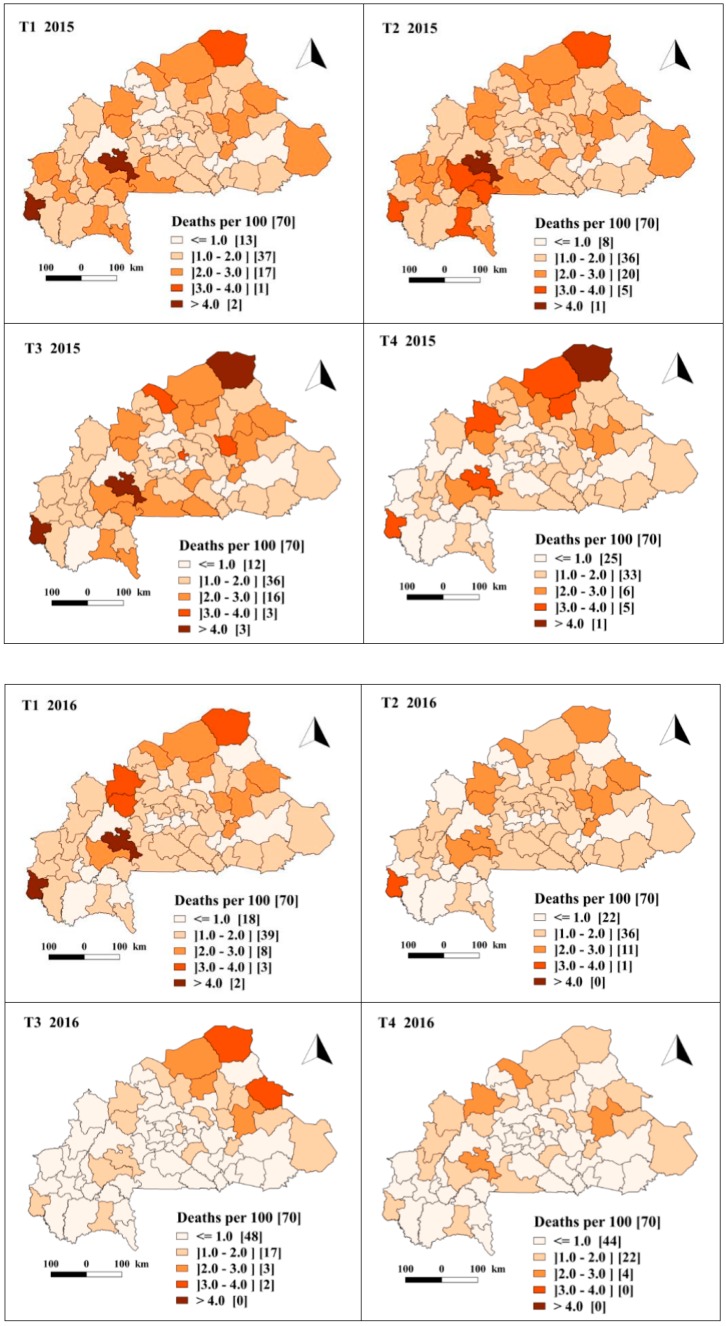

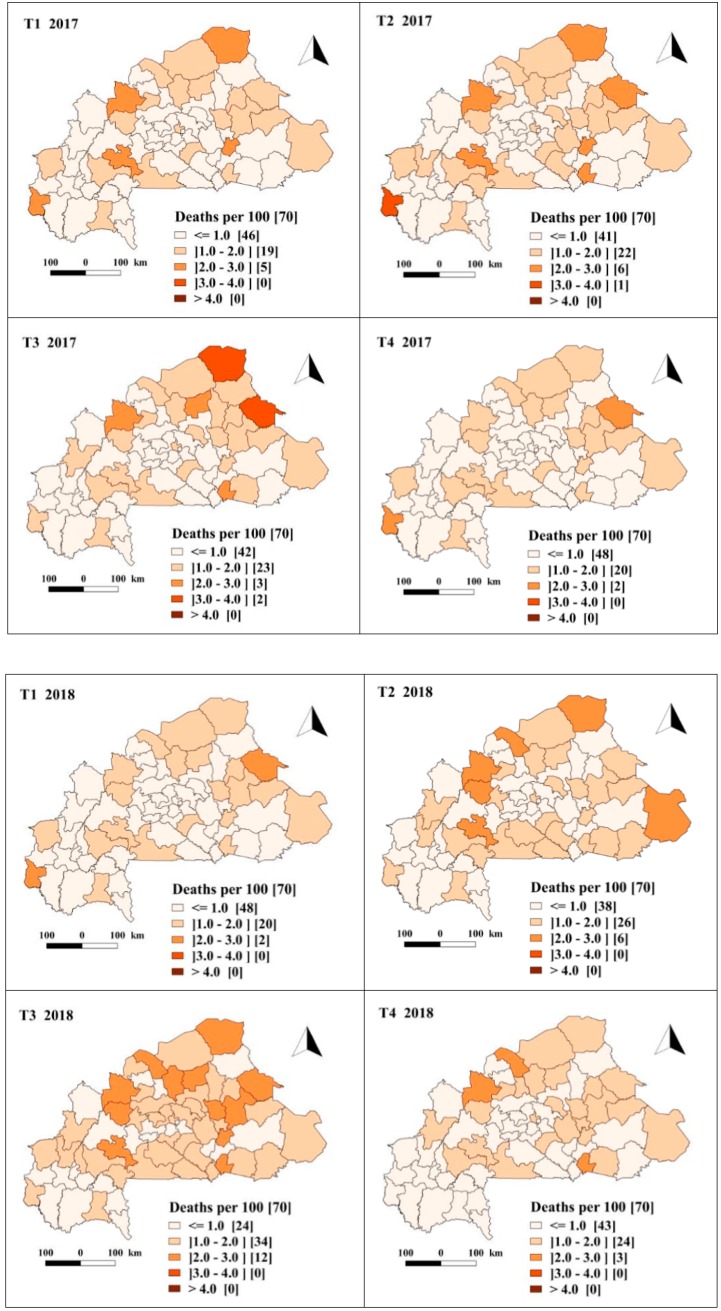
Spatiotemporal trends in the predicted malaria case fatality rate per 100 severe malaria cases in children under five, estimated from the Bayesian spatiotemporal model (January 2013–December 2018). T1, T2, T3 and T4 represent the first, second, third and fourth trimester (quarter) of each year respectively.

**Figure A1:**
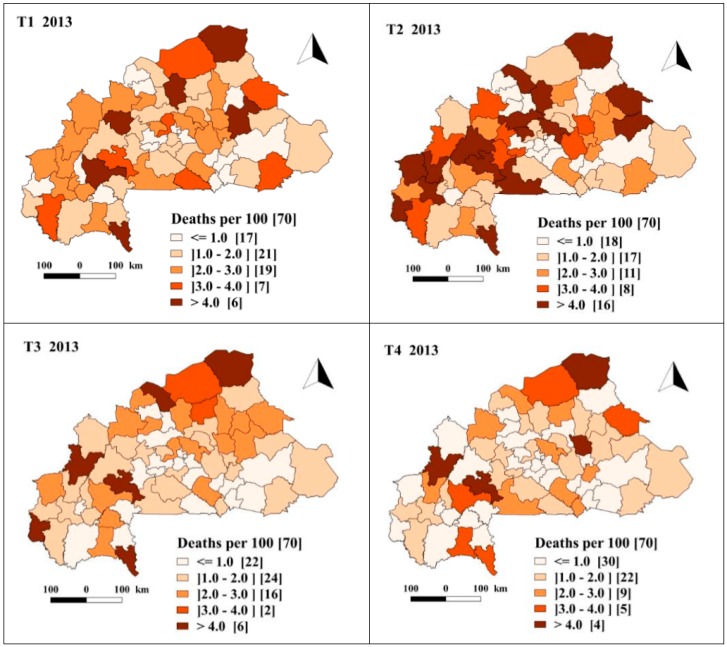

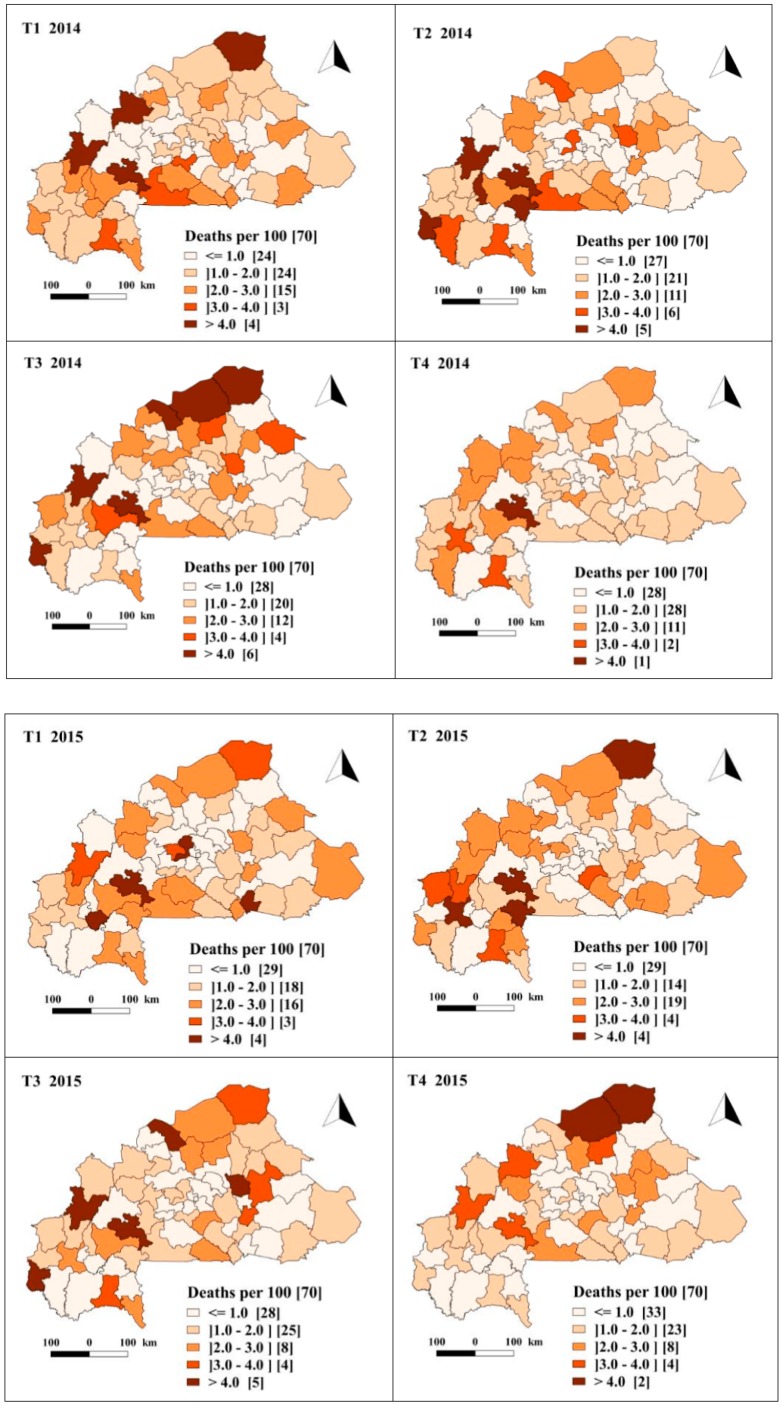

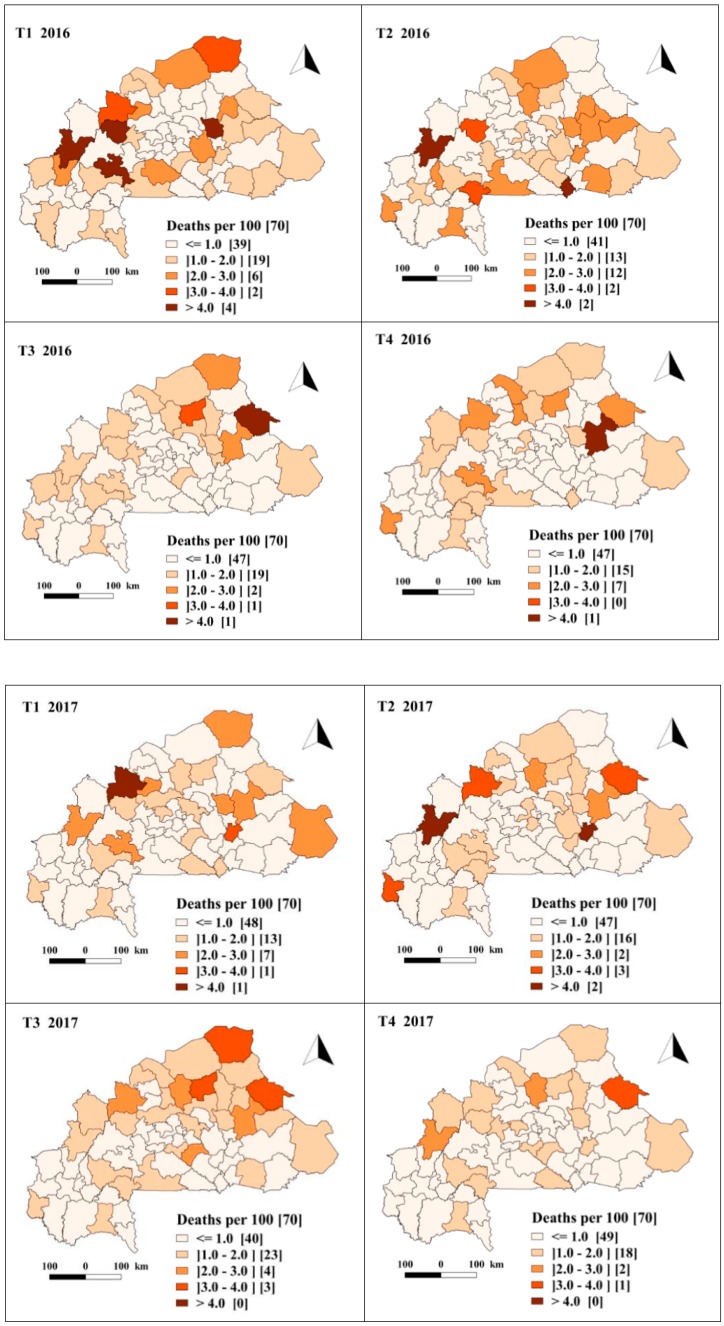

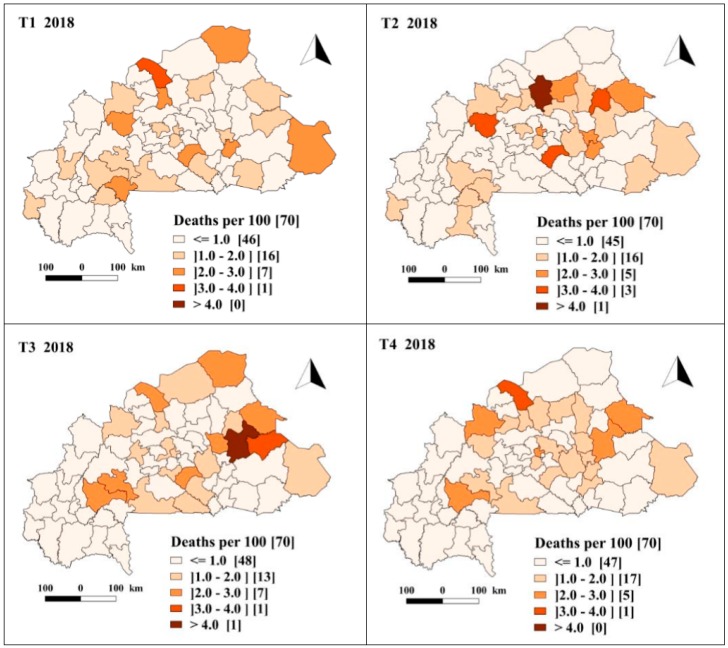
Spatiotemporal trends in the crude mCFR in children under five (January 2013–December 2018. T1, T2, T3 and T4 represents the first, second, third and fourth trimester of each year respectively.

Figure A1 (in additional file) shows the spatiotemporal evolution by quarter of the crude mCFR over the period 2013–2018, whereas Figure 2 shows the evolution of adjusted posterior mCFR. These figures show a drop in the mCFR over the period. Thus, although variable between quarters, the number of health districts with a fatality rate of malaria cases above the threshold of the national malaria control program (1.01% in 2018) fell from 40 in the fourth quarter of 2013 to 23 in fourth quarter of 2018. The adjusted fatality rate indicated the same trend with 58 health districts with a mCFR above the threshold of the national malaria control program (1.01% in 2018) in the fourth quarter of 2013 and 27 in the fourth quarter of 2018. Before July 2016, the majority of health districts had an mCFR of between 1 and 2 deaths per 100 severe malaria cases, whereas between July 2016 to December 2018, the majority of health districts exhibited an mCFR less than 1 deaths per 100 severe malaria cases. The health districts in the western (Sindou, Boromo) and northern (Gorom-Gorom) part of the country exhibited higher values with an mCFR of more than 4% between 2013 and 2016.

### 3.3. Spatial Pattern of Risk of Death Due to Severe Malaria among Children under Five

**Figure 3:**
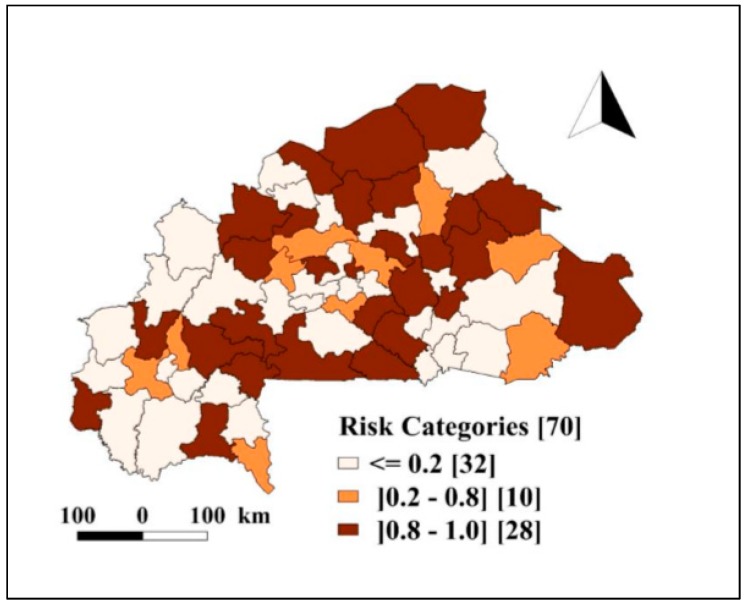
Burkina Faso health districts severe malaria deaths among children under five: exceedance probability ($\hat{P_{r}}(e^{u_{i}+\zeta_{i}}>1.01/y))$ of posterior relative risk based on Richardson’s classification. The number in bracket indicates the number of health districts within each category.

Figure 3 presents the probabilities of excess risk of death due to severe malaria based on the NMCP threshold for year 2018, which was estimated at 1.01%. The map indicated a heterogeneity in the risk between health districts and displayed the relative location of 28 identified high risk health districts. The high-risk (probabilities of excess risk) areas were predominantly in the health districts located in the northern, northwestern and southwestern parts of the country.

### 3.4. Association between Health Programs and Malaria Case Fatality Rate

Table 1 shows that the BYM full model with a temporally and spatially structured component (AR1) was more suitable than the null model to describe the spatiotemporal distribution of mCFR in children under five living in Burkina Faso. Indeed, the DIC of the full model (DIC = 17,888.29) was 108.80 points lower than that of the null model (DIC = 17,997.09). From the full model, the overall temporal variance (other the entire period) of mCFR was estimated at 1.209 (95% BCI 1.063–1.955), and the spatial variance was 1.028 (95% BCI 1.027–1.029). The space–time interaction effect was significant at 1.170 (95% BCI 1.169–1.172), indicating a heterogeneity in mCFR across the health districts over the study period.

The results of the multivariable analysis showed that a reduced risk of severe malaria death at health district level was significantly associated with the FHC policy for children under five, with the availability of mRDT and ACT and with the use of ACT for malaria treatment at health facilities. Indeed, from our findings the probability for a child under five to die from severe malaria at health facilities decreased by 50% (95% BCI 40–58%) between 2013 and 2018. In addition, a 100% increase of the proportion of health facilities with mRDT in stock was associated with a 46% (95% BCI 38–53%) decrease of mCFR, and a 100% increase in the proportion of malaria cases treated with ACT was associated with a 46% (95% BCI 38–53%) decrease of malaria case (severe) fatality rate in children under five. We also found that a 100% increase of the proportion of health facilities with ACT in stock was associated with a 50% (95% BCI 39–59%) decrease of mCFR. The distance to the nearest health facility was also significantly associated with a decrease of mCFR in children under five. However, the global acute malnutrition index and the ratio nurse/population were not significantly associated with mCFR in children under five.

## 4. Discussion

The present study is, to the best of our knowledge, the first attempt to use routinely collected malaria surveillance data to assess the contribution of control programs to mCFR changes in children under five at country level, with intra-national space–time details. Data on severe malaria cases and malaria deaths were extracted from the national health data repository (ENDOS-BF). Associations between the combined health programs and mCFR have rarely been studied previously using routinely collected data. Most studies in this area have rather focused on the effect of LLIN, the “test and treat” policy and the use of ACT for malaria case management on the morbidity/mortality of malaria [23,53,54]. Our results show a significant drop in mCFR of more than 47% in children under five between 2013 and 2018. Such a drop was previously reported in a recent study in which the aim was to map *Plasmodium falciparum* mortality in Africa between 1990 and 2015 [55]. In our study, we found that this drop was significantly associated with the launched health programs for children under five (Table 1).

It is important to note that before the implementation of FHC, several non-specific (results-based financing project, the health promotion project) or malaria-specific (seasonal chemoprevention, indoor residual spraying) control programs have been implemented for the improvement of health indicators in some health districts. These strategies have certainly contributed to the reduction of mortality among the population of children under five years of age. With the FHC policy, the test and treat components of WHO’s T3 strategy [56] are more optimal. Indeed, there was an improvement in the rate of attendance at health facilities [34] and the rate of performance of rapid diagnostic tests, enabling the detection of true malaria cases [26]. Thus, prompt diagnosis of true cases followed by effective treatment may reduce the likelihood of the progression of uncomplicated malaria cases to the severe forms responsible of case fatalities. In addition, compliance with the national malaria control program guidelines regarding the use of ACT for malaria treatment could contribute to explain this result [54].

The FHC policy may decrease mCFR by enhancing the other health interventions assessed through an increase in the use of health services, the accessibility to an early diagnostic and treatment of malaria [56,57,58,59], given that the caregivers (generally the child’s parent) are able to identify the signs of malaria in children and bring them to the nearest health facility within the 24 h of illness onset [6]. Our findings are similar to those of Posnar et al., who showed that an increased use of health services decreased malaria mortality in children under five [16]. However, studies conducted in Ghana and Cameroun did not find any significant reduction in malaria mortality associated to the FHC policy [60,61]. The authors explained their findings by the under-reporting of malaria deaths combined with a poor source document maintenance before the implementation of the FHC policy [61]. Indeed, registers were not available in many health facilities in their study. In Burkina Faso, though malaria deaths may be underestimated due to treatment-seeking behavior factors [34], underreporting is likely limited. In addition to the health facility monthly activity reports conveyed to the health statistics unit of the ministry of health, a mandatory weekly “telegram” reporting cases of core diseases of public health importance under surveillance, including malaria, is sent to the surveillance unit of the same ministry. In the end, the figures of these parallel reports must match each other. In the same study conducted in Ghana and Cameroun, the authors indicated, in addition, that an increased use of health services could potentially decrease the quality of healthcare (e.g., increased waiting time). The FHC policy should, therefore, be combined with measures to sustain healthcare quality at the risk of generating a reverse effect.

We also found that a reduction of the distance to the nearest health facility would decrease mCFR in health districts as evidenced by other studies suggesting that the shorter the distance to the health facility, the earlier malaria can be diagnosed and adequately treated [6,21].

We have also found that the effect of malaria control programs was heterogeneous across health districts. This heterogeneity could be explained by a number of logistical, medicotechnical and individual factors, varying by health district and with time. National surveys have revealed that around 21% of children who had a fever in the two weeks preceding the survey were not treated promptly [34]. This lack or delay of treatment can lead to complications resulting in death [62]. Indeed, it has been reported that in severe malaria, neurological manifestations appear within the first 12 h of admission [63] and is fatal without treatment [64]. Severe malaria treatment implies, in addition, supportive care to control complications of especially poor prognosis factors such as severe anemia, respiratory distress, bacterial co-infections and acute renal failure [65,66,67,68,69,70]. Blood, oxygen, dialysis and mechanical ventilation [71] may be necessary for an appropriate treatment of severe malaria. The health districts are uneven vis-à-vis the availability of these means for supportive treatment. Finally, other poor prognostic factors such as acute malnutrition [62,72] cannot be suppressed in an emergency. In addition, the disparities between health districts observed in the present study could be partly explained by other health programs. Indeed, the community-based health workers program was implemented in 2015 countrywide. Community-based interventions were reported to increase the coverage of most malaria control interventions [73].

Most studies assessing the impact of combined effect of health interventions (FHC, “test and treat” and use of ACT for malaria case management) on mCFR in children under five do not examine the spatiotemporal variability of the risk at the intra-national level [16,23,53,54,60,61]. It is often postulated that the efficacy of national malaria control programs relies essentially on the availability of reliable local health statistics to guide the decision-making process [74]. Our results confirm this assertion and have implications for the control of mCFR in children under five. Indeed, the measurement of the association with the combined effect of health interventions (FHC, “test and treat” and use of ACT for malaria case management), the identification of health districts with intermediate to excess risk and CFR could guide the planning of control interventions by the NMCP. In Burkina Faso, health districts are the operational level for the implementation of health policies, and one of their missions is to implement malaria control interventions [75]. Such interventions include ensuring availability of mRDT at health facilities and at the community level, the availability of ACT and injectable artesunate for the treatment of uncomplicated and severe malaria, the provision of health facilities with LLIN for free distribution to target populations as well as population information, education and communication (IEC) for an optimal use of these LLIN [53,56,57,58]. In the short term, the NMCP could reinforce the IEC for malaria control at the community level in health districts with moderate to high risk. Such a targeted and timely response from the NMCP relies on its capacity to collect, analyze data and generate situation maps in real time.

In the present study, we did not find an association between global acute undernutrition and mCFR. This result is in keeping with the findings of some prior studies [76,77,78,79]. The association between nutritional status and malaria, in the current state of evidence, remains quite fuzzy. Clinical malnutrition is described as a heterogeneous group of nutritional disorders, and its interplay with immunity and infection is complex [80]. Impaired immunity is likely a consequence as well as a cause of malnutrition [81]. While an increased risk of infection can be observed in some nutrient deficiencies, a protective effect is observed in others and, at last, an increased risk or protective effect can be observed in some other deficiencies depending on the context [82]. Earlier studies reported or suggested a protective effect of undernutrition against malaria, especially refeeding in famine context [83,84,85,86], or reported no association between malaria and undernutrition. However, there is now a strong body of evidence and general agreement that undernutrition, though it may not have a strong effect on malaria infection or incidence, can increase the illness severity and worsen severe malaria outcome [87]. The risk of death was associated with severe acute undernutrition in a number of studies including hospital-based [88,89] ones and randomized trials [90]. Some of these studies did not distinguish between wasting and stunting [91]. In a large multisite, multi-country hospital-based study, the z-score was linked to early, intermediate and late malaria mortality globally, and severe acute malnutrition was associated with mortality at six out of eight sites [92]. The wide array and disparities in the methods used limited the feasibility of conclusive meta-analyses.

The present study has limits. First, the number of severe malaria cases and malaria deaths could have been underestimated because of the low use of health services. In addition, the CAR models we used are subject to estimation biases due to ecological error [93]. However, the models we used took into account the uncertainty in the estimates and the biases related to the data sources allowing us to generate robust estimates.

## 5. Conclusions

The present study assessed the association between malaria control programs (free-of-charge healthcare, test and treat, use of ACT) and mCFR in children under five living in Burkina Faso. Our findings showed a decline in mCFR and an association with control interventions. Moreover, our results showed geographical differences in the mCFR decline over time in Burkina Faso. Malaria case fatality rate tended to increase in some health districts. If this tendency is maintained in the same localities in subsequent evaluations, they would then be worthy of thorough investigation to identify and address the factors impeding the policy. Moreover, in a resource-limited context, our findings could guide the NMCP in hierarchizing the health districts for the implementation of appropriate control interventions. In addition, our findings highlighted the necessity to sustain and strengthen the health programs in effect to further reduce malaria deaths in Burkina Faso. Finally, the model could be useful to other countries for the assessment of the effect of malaria control interventions and to Burkina Faso for periodic re-evaluation of these programs.

## 6. Research Data and Supplementary Materials

The dataset of severe malaria cases and malaria death cases aggregated at the health district level is available at the Directorate General of Studies and Sectoral Statistics of the Ministry of Health in Burkina Faso.

## Figures and Tables

**Table 1:** Association of health programs with severe malaria case fatality rate estimated from the spatio-temporal model adjusted by contextual factors.

Predictor	Adjusted IRR (95% BCI)
Free of charge health care	
No	1
Yes	0.47 (0.38, 0.58)
Availability of mRDT ^a^	0.54 (0.47, 0.62)
Availability of treatment ^a^	0.50 (0.41, 0.61)
Use ACT for treatment ^a^	0.54 (0.47, 0.62)
Global acute malnutrition	
<10%	1
≥10%	1.06 (0.99, 1.13)
Distance (km) ^b^	
<5	1
≥5	1.12(1.03, 1.22)
Population nurse ratio	
<5000	1
≥5000	1.07 (0.99, 1.15)
**Spatial variance**	**Mean [95% BCI]**
Non-structural Variance	1.036 (1.035, 1.037)
Structural Variance	1.028 (1.027, 1.029)
**Temporal variance**	**Mean [95% BCI]**
Structural variance	1.209 (1.063, 1.955)
Interaction variance	1.170 (1.169, 1.172)

^a^ Coverage (availability of malaria rapid diagnostic test (mRDT) and treatment) and use of artemisinin-based combination therapy (ACT) for treatment were modelled on a scale of 0 to 1; therefore, a one unit increase in coverage corresponds to a 100% increase, which implies a shift of the current value by 100%. ^b^ Distance to the nearest health facility.

## Abbreviations

BCI	Bayesian credible intervals
DIC	Deviance information criterion
DHIS2	District Health Information System
INLA	Integrated Nested Laplace Approximation
mCFR	malaria case fatality risk
RDT	Rapid Diagnostic Test

## Appendix A1. Estimating District-Level Interventions Coverage

The data on the availability of kits for the rapid diagnostic test (mRDT) for malaria, the availability of treatment for the management of malaria cases and the use of ACTs for the treatment of malaria used in this study come from periodic Survey on Availability and Readiness Assessment (2012, 2014, 2016) and Malaria Indicators surveys (MIS) for year 2014 and 2017. These data come from national survey designed to have a regionally representative estimate. A conditional autoregressive model (CAR) formulated in a Bayesian framework with a binomial distribution was performed to estimate indicators at the district level.

Let $Z_{j}$ be the number of health centers with mRDT kits in district j = 1, …, 70 and $M_{j}$, the total number of health centers sampled and interviewed in district j. We assume that $Z_{j}$ follows a binomial distribution, namely $Z_{j}|M_{j},\pi \left(j\right)\sim \mathrm{Bin}\left(M_{j},\pi \left(j\right)\right)$ ∀ j = 1, …, 70, where π(j) is the proportion of health center having a kit for the test in district j.

(A1) $$\mathrm{logit}\left(\pi \left(j\right)\right)=\beta_{0}+\omega_{j}$$

where $\beta_{0}$ is a constant and $\omega_{j}$, ∀ j = 1, …, 70 are modeled via a CAR prior. Each $\omega_{j}$ conditioned by the neighbor $\omega_{k}$ follows a normal distribution with an average equal to the average of the neighboring districts $\omega_{k}$ and a variance inversely proportional to the number of neighboring districts $m_{j}$, i.e., $\omega_{j}|\omega_{k}\sim N\left(\gamma \underset{l\in \delta_{j}}{\sum }\;\omega_{k},\;\frac{\sigma_{\omega }^{2}}{m_{j}}\right)$, where γ quantifies the magnitude of the spatial correlation present in the data, $\sigma_{\omega }^{2}$ measures the spatial variance. $\omega_{j}$ and $\omega_{k}$ are adjacent districts in the set of all adjacent districts $\delta_{j}$ of district j, and $m_{j}$ is the number of adjacent districts.

We assumed noninformative Gaussian distributions prior for $\beta_{0}$~ N (0, 102). A gamma distribution prior for $\sigma_{\omega }^{2}$~ Ga (0.1, 0.001).

We have use similar formulations for the availability of treatment in health centers and the use of artemisinin-based combination therapies (ACTs) for treatment in households.

## Appendix A2. Choice of ZIP Model or ZNIB for Modelling

We performed statistic investigations to raise the over dispersion in the data. We concluded that there was an over dispersion in the data. Since the overdispersion was due to an excess of zero, a ZIP or ZNIB was suitable to handle this issue. To choose between ZIP or ZNIB we proceeded as follows.

It appeared from the two analyzes that the DIC of the ZIP was lower (DIC = 17,888.29) than the ZNIB model (DIC = 18,343.52). We then selected the ZIP model for our study.
